# Supplementary material for: Protocol for a pragmatic cluster randomised controlled trial to evaluate the effectiveness of digital health interventions in improving non-communicable disease management during the pandemic in rural Pakistan
Source: PLoS One. 2023 Oct 10;18(10):e0282543. doi: 10.1371/journal.pone.0282543 (PMC10564142; doi:10.1371/journal.pone.0282543)
Supplement: S2 Checklist — (DOCX) [file pone.0282543.s002.docx]

**S2 Checklist. Inclusivity in global research questionnaire**

**Ethical considerations, permits and authorship**

*This section is applicable to all research types.*

Provide details as to who granted permissions and/or consent for the study to take place in the Methods section of your manuscript. This should include the names of **all** ethics boards, governmental organizations, community leaders or other bodies that provided approval for the study. If individuals provided approval refer to these people by their role or title but do not list their name(s).

Reported on page number: 7

If there were any deviations from the study protocol after approval was obtained please provide details of these changes in the Methods section of your manuscript.
Did this study involve local collaborators that are residents of the country where the research was conducted or members of the community studied? If you do not have any authors from said communities, please provide an explanation for this below.

Reported on page number: N/A

The Association for Social Development (ASD), a non-government organisation (NGO) based in Pakistan, is the leading partner in project design and implementation.

Everyone listed as an author should meet PLOS’ criteria for authorship and all individuals who meet these criteria should be included in the author byline, rather than the acknowledgements. For further information please see the journal’s Authorship Policy.

**Human subjects research (e.g. health research, medical research, cross-cultural psychology)**

Did you obtain written informed consent from a representative of the local community or region before the research took place? How did you establish who speaks for the community? Details of written informed consent obtained from study participants should be reported separately in the Methods section of your manuscript.

Informed consent forms with patients’ preference of language (Urdu or English) will be collected before any patient is recruited into the study. The healthcare providers will fully explain the information sheet and consent form to the participant in Urdu or English. Both the participant and the healthcare provider are required to sign the consent form. If the participant is not able to sign due to illiteracy, they can use their thumbprint instead. The healthcare provider will keep the signed informed consent form and leave a copy to the participants for their reference.

How did members of the local community provide input on the aims of the research investigation, its methodology, and its anticipated outcome(s)?

Local community members were interviewed during the situation analysis phase. The input was incorporated in deisgning the trial phase.

When engaging with the local community, how did you ensure that the informed consent documents and other materials could be understood by local stakeholders?

The consent forms will be translated in local language, Urdu, and will be explained by the health provider in the process.

Will the findings of the research be made available in an understandable format to stakeholders in the community where the study was conducted (e.g. via a presentation, summary report, copies of publications, etc.)? Please provide details of how this will be achieved.

The trial data will be analyzed in Pakistan and Canada to produce reports and papers. We will provide feedbacks to patient community using public education message when the project outcomes are ready. We will coordinate with Department of Health Services at the provincial and national levels to translate evidence into program decisions for incoperating mobile technologies into routine NCD care in Punjab, other provinces of Pakistan, as well as adapting the research evidence for practice in other LMICs. We will publish the results in leading academic journals and provide policy briefs to policymakers.

**Non-human subjects research using specimens/ animals collected as part of the study, or those housed in archival collections. Examples include archaeology, paleontology, botany and zoology.**

Did the permission you obtained from a local authority to perform the study include an agreement on access to outputs and benefit sharing? This may include procedures to enable fair distribution of the benefits and resources arising from the research performed. Please include any details of Prior Informed Consent and Benefit Sharing Agreements obtained. These may be required by field-specific regulations, for example the Convention on Biological Diversity (CBD) and the associated Nagoya Protocol.

N/A

If the material used in your study was imported, please A) provide the year it was imported and B) indicate whether permits were obtained to import/export the materials used, C) provide details of any permits obtained. If this information is not available, please indicate this.

N/A

If you used archival specimens, please state how the material used in your study was acquired by the institute it is held in and provide details of any permits obtained for the original excavations/ sample collection. If this information is not available, please indicate this.

N/A

How was the potential cultural significance of the materials collected in your study to local communities considered in your research design? Were Indigenous peoples and/or local researchers and institutions involved with archaeological excavations / collection of specimens? If so, please provide a description of their involvement.

N/A

If your manuscript includes photographs of human remains please indicate whether authors obtained permission from descendants or affiliated cultural communities to do so.

No pictures are included in the manuscript.
