# Supplementary material for: Protocol for a pragmatic cluster randomised controlled trial to evaluate the effectiveness of digital health interventions in improving non-communicable disease management during the pandemic in rural Pakistan
Source: PLoS One. 2023 Oct 10;18(10):e0282543. doi: 10.1371/journal.pone.0282543 (PMC10564142; doi:10.1371/journal.pone.0282543)
Supplement: S1 File — (PDF) [file pone.0282543.s003.pdf]

Title: Implementing a telemedicine and remote-mentoring platform to provide integrated noncommunicable disease and COVID- 19 care in primary care facilities in rural Pakistan

1. Background: The World Health Organization (WHO) recently summarized gaps of COVID-19 challenges,[1] including lack of a national plan to confront and control the pandemic, poor health financing, politicizing proven infection control strategies; and non-optimal use of telemedicine for supporting practices. The report also states challenge of effective control of non-communicable diseases (NCDs) during the pandemic in low-and-middle-income countries (LMICs).[2] People with diabetes and hypertension are older, often over-weight, and have different comorbidities. [3] They are at greater risk of severe complications from COVID-19,[4] but on the same time they require continuous care, which has now been made more difficult due to infection control and social distancing measures at health facilities.[5] A WHO survey in May 2020 showed that over half of its 155 member countries had disrupted hypertension, diabetes treatment and cardiovascular emergencies. Majority of countries responded that staff for routine health programs such as NCDs were partially or fully reassigned to support COVID-19.[6]

Telemedicine and virtual care including telephone and video consultation on mobile phones, have been used as tools to ensure continued health care globally during the COVID pandemic. Even before the COVID-19 pandemic, many have emphasized that digital health tools are important for the prevention and management of NCDs as digital health tools play an essential role in integrated care, especially in managing multimorbidity delivering precision medicine, and increasing medication adherence.[7] WHO reported that 58% countries began to adopt telemedicine in their NCD services,[6] but the evidence is mostly reported from US, China and Iran.[8] The most common forms of telemedicine were phone calls and social media platforms, with some been connected to electronic medical records (EMR). Telemedicine is an effective strategy in reducing exposure to COVID, nosocomial and community transmission, and is acceptable to health providers and patients.[9] However, most primary care providers in LMICs lack the capacity to develop qualified telemedicine platforms.[10] Only a small proportion (5%) of patients with diabetes in India used telemedicine (i.e., simple calls) during the lockdowns, but their response was overwhelmingly positive.[11] Primary-level screening services, delivered through fixed/mobile primary healthcare clinics, community health workers or health campaigns and mobile consultations were identified by health workers, managers and community residents for hypertension in Kenya and Nigeria.[12]

Pakistan ranks 7th globally amongst countries with the highest number of persons living with diabetes and hypertension.[13] It has gone through three significant COVID waves and is currently into its fourth wave.[14] Patients with diabetes and hypertension in Pakistan are inadequately managed, as evidenced by high rates of vascular complications and low blood pressure (BP) control rates (30%).[15,16] Pakistan responded to the COVID epidemic with a lockdowns and focused on hospital-based inpatient care for those with severe symptoms, a strategy that would not reduce community transmission, as shown in other countries.[17] Pakistan also saw a disruption in access to routine care with outpatient visits in **Rural Health Centres (RHCs)**, i.e., the rural public primary facilities, declining by 40% in June 2020.

According to Pakistan Telecommunication Authority, there are 185 million mobile connections (84% tele-density) in Pakistan among a total population of 225 million; and around half of these connections are smart phone users. The internet broadband subscribers stand at 104 million with a penetration of 48%.[18] With a digitalization at an early stage especially in rural areas, Pakistan has seen several telemedicine and mHealth initiatives piloted by organizations such as COMSTATS, and Aga Khan University Hospital Karachi. COMSATS has established telehealth clinics in isolated areas to provide easy access to healthcare for the most vulnerable population of the country.[19] However, none of them has been able to sustain and successfully scale to a large population.

Though unprecedented progress has been made in vaccine development, future of the pandemic remains uncertain given that the majority of LMIC population will not be vaccinated in the several years, while new SARS-COV-2 variants are emerging rapidly. The pandemic is likely to become endemic for years to come in LMICs.[20] Many countries are working on strategies to minimize the COVID-19 transmission and provide vaccination. Pakistan does not have the resource to fully vaccinate its entire population. Therefore, there is an urgent need to strengthen primary care to screen, triage and manage NCDs and at the same time to provide home and community-based solutions to prevent community

transmission of COVID.[10] In the context, care for patients living with diabetes/ hypertension must address there COVID needs, while continuing NCD care with minimal transmission risk. This needs increased skills of health providers in identification and diagnosis of NCD cases and improved triage, diagnosis, treatment and referral services for NCD in intervention population. A remote training and monitoring platform is equally important to maintain high quality of care in LMICs. Our previous studies demonstrated the feasibility and effectiveness of a community-based hypertension/ diabetes management in Pakistan.[21,22] The Extension for Community Healthcare Outcomes (ECHO), a remote training and mentoring system, was proved effective to improve NCD management.[23] We have successfully conducted online training of COVID-19 in primary healthcare facilities in Asia and Africa.[24,25] There is a high need in LMICs for evidence regarding the feasibility and effectiveness of an integrated COVID-NCD prevention and care that is guided by implementation science frameworks.

2. Hypothesis, Aims and Objectives: We aim to design a package of integrated Covid-NCD care in RHCs of Punjab Province, Pakistan. Based on our existing NCD projects in Punjab, we will adapt our COVID-19 primary care guidelines developed in other LMICs,[24] to integrate COVID-19 vaccination, health education, case management and support for patients with hypertension/ diabetes. Our study is guided by the implementation science RE-AIM framework, which tackles health system factors at multiple levels.[26,27] Our overall aim is to reduce patient overload at hospitals, avoid nosocomial transmission, reduce community transmission, and provide patient support.

Specific objectives include**:** 1) to conduct a rapid context analysis at primary care level by focusing on issues relating to how to embed COVID-19 care into the existing NCD programs in RHCs; 2) to evaluate the effectiveness of the integrated COVID-NCD care package based on clustered randomized controlled trial; 3) to conduct process evaluation and cost-effectiveness analysis that is embedded in the project to facilitate future scaling up the evidence in Pakistan and other LMICs; 4) to plan, prepare for scale-up the package in Pakistan and other LMICs.

3. Applicants: The team has expertise in public health, clinical care, biostatistics, social science and information technology. The Nominated Principal Applicant (NPI), Xiaolin Wei, is a clinical public health expert with extensive experience in leading implementation science including cluster randomized controlled trials in diabetes/ hypertension,[28] tuberculosis,[29] and antimicrobial resistance[30] in LMICs. In addition, he leads a CIHR/IDRC funded study to develop COVID-19 guidelines in the Philippines and Sri Lanka. He will oversee all aspects of the study and ensure its completion together with the principal applicant, Mohammad Amir Khan from ASD in Pakistan. Khan is the Founder and head of ASD. He has led scaled implementations of disease control interventions funded by the Global Fund, UK DFID and Grand Challenge Canada. He leads a World Diabetes Federation (WDF) grant to implement decentralized NCD care in 84 RHCs in Punjab. He recently completed two trials in diabetes,[31] and hypertension[22] management. Haroon Jehangir Khan, the Director General of Health Services Punjab, Pakistan, is the principal knowledge user of the project. He will provide political, administrative and logistic support to project design and implementation and will lead the knowledge translation in Pakistan. Dure Technologies a digital innovation enterprise headquartered in Geneva, Switzerland will be explored to develop patient engagement application to support patient: a) knowledge seeking; b) challenge reporting; c) peer-supporting; and d) (group) tele-consultation.[32] It offers a wide variety of features including live video conferencing, patient management, store & forward communication, appointment management and compatibility with almost all diagnostic devices. Other co-applications in Canada represent a balanced team with researchers from different career stages, covering diabetes and hypertension care (Gary Lewis), process evaluation (Warren Dodd), health economy (Audrey Laporte), computer science (Jim Wallace), spatial analysis (Erjia Ge) and two PhD student trainees (Victoria Haldane, for qualitative study and gender analysis, and Hammad Durrani for implementation). Our collaborators include 1) John Walley a clinical professor of international public health at the Nuffield Centre, University of Leeds, who has collaborated with Wei and Khan for more than 20 years on TB, HIV, and NCD in Pakistan and China. Their joint work has evolved into an “embedded research and service development approach” linking research activities with program implementation plans and challenges;[33] and 2) Bruce Struminger, Senior

Associate Director of the ECHO Institute at the University of New Mexico Health Sciences Centre. He will provide in-kind support in adapting the ECHO-training platform to support telemedicine.

4. Study Phases and Methods

4.1 Study Settings: The Province of Punjab, Pakistan, has 110 million inhabitants in 36 districts; about two-third of the population live in rural areas. The rural population is served mainly by a network of more than 315 RHCs, and 120 district level hospitals. Each RHC provides primary care to a catchment population of 200,000 rural inhabitants. RHC staff includes: 1) three or more doctors; 2) five or more allied health workers including paramedics, lady health visitors, nurses, dispenser, laboratory technician; and 3) support staff. Currently, the research team in Pakistan ASD is leading the WFD project in 84 RHCs among 10 districts in Panjab. The WRF project have diagnosed and are providing treatment to 10,000 patients with diabetes or hypertension. Currently, no Covid-19 care or prevention is provided in RHCs. Testing is only provided at the district hospital which is remote to most patients.

4.2 Phase I: Context analysis and intervention design (objective 1), 3 months: We will mainly use document review and qualitative study to understand the impact of the pandemic, assess bottlenecks, any resilience of the system that helps tailor possible approaches. This will be conducted in 2 RHCs based on their level of performance (good or not-well) as recommended by ASD, one as intervention and another as control. Based on this, we will develop the interventions.

Study methods including:

1) document review: to review current NCD-COVID care under the routine WDF project, and existing national and global guidelines for hypertension, diabetes and COVID care.

2) stakeholder meetings: with relevant government health decision makers, NGOs and patients, healthcare providers at RHCs to assess the current state of hypertension and diabetes prevention and care, and to share objectives and components of study interventions.

3) key informant interviews: We will conduct in-depth interviews with key stakeholders. These are likely to include leaders, policymakers, health providers from RHCs, the district, and community. We will explore the structural, organisation, provider and patient factors that may determine NCD care implementation, and how NCD care is affected/interrupted by the pandemic. We will also explore how sex and gender factors such as cultural factors, as well as gender-based inequities or barriers to care, are relevant in current NCD care under the pandemic. Exact numbers of interviews will be decided based on further understanding of the context. Phase 1 will also be used for ethics approval, modifications of sample size estimation and staff recruitment for phase 2 if necessary. If the interventions are not to be revised substantially, these two pilot sites will serve in the trial (internal pilot).

4.3 Phase II: Implementation and Evaluation (objective 2-3), 12 months implementation:
We will conduct a parallel two-arm, cluster randomized controlled trial of the enhanced NCD-COVID-19 integrated care package in rural Punjab, Pakistan. The Province of Punjab has 110 million inhabitants in 36 districts; about two-third of the population live in rural areas. The rural population is served mainly by a network of more than 315 rural health centers (RHCs), and 120 (sub)district level hospitals. Each rural health centre provides primary care to a catchment population of 200,000 rural inhabitants. The staff of RHC includes: 1) three or more doctors; 2) five or more allied health workers including paramedics, lady health visitors, nurses, dispenser, laboratory technician; and 3) support staff. RHCs offer diagnostic and treatment services for communicable diseases, obstetric and neonatal care, child health, malnutrition and NCDs. The Directorate General Health Services Punjab has already scaled integrated diabetes-hypertension care at the district hospital level, including diagnosis, treatment, lifestyle counselling and reporting tools – using a package of care developed with ASD and Leeds. Currently, ASD is leading the WDF project in 75 RHCs among 10 districts in Punjab to offer NCD care which is focused on diabetes-hypertension diagnosis and treatment.

4.3.1 Randomization and blinding: We will recruit and randomize 30 out of the 75 RHCs which currently offer hypertension-diabetes care. RHCs, stratified by district, will be randomly allocated into

intervention and control at 1:1 ratio. RHCs will not be blinded due to the nature of the intervention. To mitigate the risk of bias, we will: 1) mask the statistician and the Data Safety Monitoring Board (DSMB) when data is presented and analyzed; and 2) employ objective outcomes.

4.3.2 Inclusion and exclusion criteria: We will invite all clinically diagnosed persons living with hypertension (SBP> 140 mmHg or DBP >90 mmHg) who 1) reside in the selected RHCs, 2) are 25 years or older, 3) and provide informed consent, and 4) have a smartphone or can access to a smartphone from a relative..

4.3.3 Our usual care will be enhanced care under the WDF project, where the NCD (hypertension/ diabetes) care package is implemented. The WDF project provides Zoom-based training of NCD care to RHC staff, but not telemonitoring and mentorship. The NCD care is delivered through outpatient consultations where patients must visit RHCs to get screened, diagnosed, registered, and treated. Since August 2020, regular visits to RHCs have been largely resumed. Medicines are provided freely in RHCs or purchased from private pharmacies if not available. Patients are referred to district hospitals for cardiovascular disease or COVID diagnosis if suspected. Under the usual care, patients with diabetes/hypertension are required to visit RHCs every month and have their health records recorded entered in RHC's EMR. None of the components in an enhanced NCD-COVID integrated care-package, neither the telemedicine, the ECHO-based tele-mentoring platform, nor peer support, will be implemented in control facilities.

4.3.4 The intervention package of care contains:

- **Integrated NCD-COVID guidelines** to support RHC staff to deliver COVID care by triaging symptomatic patients; promoting rational use of personal protection equipment (PPE); promoting COVID vaccination; replacing face-to-face follow-ups by virtual consultation; and providing case management, health education, and home quarantine support. We will adapt our LMICs COVID-19 guideline into Pakistan to be embedded in the NCD (diabetes/ hypertension) care in the WDF project.
- **A telemedicine platform** containing a smart phone app component, to link patients with facility staff and community peers (champions). The technology will offer patients: a) access to knowledge on NCD and COVID care, including healthy lifestyle and behaviours to prevent COVID transmission; b) report care challenges being faced at respective RHCs (or at the referred facility); c) get peer-support (through technology assisted audio tele-conference of patient groups) for practicing recommended behaviours at home/ communities; d) get routine NCD follow-up advice; also seek advice on medical complaint/ condition. We will also explore the feasibility of using auto-generated messages through the digital application.
- **An ECHO based online platform** for staff training, mentoring and performance monitoring will be employed for case-based learning using best practice and data. RHC staff will be trained in screening, prevention and integrated management of Covid-19 and NCD, as well as how to use the mobile app and send health promotional SMS messages. Based on our experience in the Philippines and Sri Lanka, we will provide training to RHC staff and community health workers regarding how to improve vaccine confidence among high-risk groups for COVID-19, including NCD patients.

Patients will receive: 1) the option of attending virtual consultation with health providers in RHCs through smart phones, SMS or calls; 2) targeted health education for NCD and COVID; 3) hypertension and diabetes follow-up care through the telemedicine platform; and 4) COVID care and support through telemedicine. Patients who do not have access to smart phones will be helped by family members. We will also identify "patient champions" who are persons with lived experience of well managed diabetes, hypertension or COVID in the catchment area and are willing to provide consultation support to patients in the same area through the telemedicine platform. Creation of study materials and intervention components will be grounded in a gender and culturally responsive approach, informed by local stakeholders and best practices, to support equitable delivery of services and workforce capacity building.

4.3.5 Outcome evaluation: We will assess effectiveness through the following indicators measured at 10 months. Our **primary outcome** is the average systolic blood pressure (mmHg) measured in the RHC once a month of the last three months. Our secondary outcomes include: 1) the average diastolic blood pressure (mmHg) measured in the RHC once a month of the last three months; 2) percentage of patients with controlled BP measured below 140/90 mmHg for patients with hypertension but without diabetes, and

below 130/80 mmHg for patients with diabetes. The denominator will be all NCD participants in RHCs. The numerator will be patients with controlled BP, measured at 10 months; 3) random blood glucose (mmol/L); 4) body mass index (BMI); 5) attendance rate to monthly meetings with RHC doctors (face-to-face or digital); We will also measure indicators related with COVID-19: 6) proportion of patients who have had at least two COVID vaccinations; 7) proportion of patients reporting COVID symptoms; 8) proportion of patients who received COVID care (diagnosis, treatment or home support) from RHCs among those reported COVID-19 symptoms; and 9) proportion of participants been admitted in hospitals for any reasons; 10) mortality rate of participants for any reasons during the 10 months of follow up. We will collect blood pressures, blood profile, and patient body weight and height at Month 1 and 10in the RHCs. Participants will be asked for fasting for 24 hours and provide a blood sample. During the consultation, the RHC doctor will measure the participants' BP using a calibrated electronic sphygmomanometer after 5 minutes of seated rest, and measure body weight and height. Blood samples will be collected and analyzed in RHC according to national guidelines for fasting plasma glucose. We will collect attendance rates using the routine patient treatment cards in RHCs to record both physical and virtual consultations. We will conduct a survey at the recruitment to collect patient demographics, socioeconomics, smoking status, any COVID symptoms of themselves or family members, health service use, and quality of life. The survey will be repeated at Month 10 in the last follow-up to collect COVID-19 vaccination, symptoms and related care utilization.

4.3.6 Sample size and justification: Sample size is based on demonstrating the intervention achieves a non-inferior result in systolic blood pressure (SBP). We will define a non-inferiority margin of 2.5 mmHg. The reasoning behind this is that SBP can be quite variable over the day, easily ranging 5 mmHg. Thus, if the intervention results in SBP no worse than control by 2.5 mmHg, it would represent non- inferiority. Based on prior work, [34,35] , the standard deviation of SBP is expected to be about 11 mmHg. For an individually randomized trial, a sample size of 332 per group would have 90% power with a one- sided type one error of 5% to demonstrate non-inferiority at a 2.5 mmHg margin. We will aim to recruit 40 patients per cluster. If we assume a modest intra-class correlation (ICC) of 0.03, indicating a sample size of 614 per group to achieve the same power. Thus, 15 clusters per group of 40 patients each will be required to obtain the minimal required sample size. Considering a 20% loss-to-follow-up rate based on our trials in Pakistani[21] and China,[28] we aim to increase to 50 patients per cluster, that is a total of 1500 patients to be recruited. Power will increase if all clusters meet the enrollment target of 50 patients; furthermore, the impact of averaging each patient's systolic blood pressure over the last 3 months should reduce the standard deviation, further increasing power. The reason for averaging is that single systolic blood pressure measurements are less representative of blood pressure.

4.3.7 What is the planned recruitment rate?

Each RHC has at least 100 registered patients with hypertension/ diabetes. We anticipate recruiting 50% of them, to generate 50 participants. The recruiting process will take less than two weeks as all are existing patients registered in RHCs.

4.3.8 How will source of bias and risks be taken account of?

Potential sources of bias include 1) that this is an open label trial; and 2) non-compliance of health providers and patients. We have described measures to mitigate the risk of an open label trial in 4.2. We will provide ECHO-based remote training and supervision with health providers to identify non-compliance and provide timely feedback. Patient champions will connect patients or their relatives who have low literacy in smart phones or do not attend virtual consultations. In addition, we will provide gender sensitive support. The key risk is change in provincial level commitment of the NCD Prevention and Control Program due to staff turnover or other events. The mitigation strategy includes to: 1) document the relationship; 2) keep the NCD Program continuously engaged; and 3) continue the technical assistance for sustained public funding.

4.4 Process Evaluation: We aim to describe health system and service delivery context in which the interventions are implemented, explore delivery and dose of intervention components received, and understand mechanisms of impact and unanticipated consequences on health providers and parents. This

fits into the qualitative indicators set up in the RE-AIM dimensions.[26] Methods will include document review (e.g., meeting minutes), observations of training sessions, and qualitative interviews with stakeholders. Specifically, we aim to understand: 1) *Reach*: the recruitment process and the program inclusion; 2) *Effectiveness (feasibility and acceptability)*: to what extent our measures are understood and accepted by patients; 3) *Adoption*: changes of interventions made at the facility level; how the NCD-COVID care connects with other programs such as tuberculosis and immunization; and any resistance from structural, professional or personal factors in adopting the change; 4) *Implementation:* issues related to fidelity/quality of implementing the NCD-COVID program, and any unanticipated consequences; 5) *Maintenance:* to what extent the NCD-COVID program is institutionalized in different RHCs. We will also conduct spatial analysis to understand how patients travel time to RHCs may affect their care utilization and BP control levels. We will conduct a mixed methods process evaluation using program record review, interviews, and observations, with a broad section of patients, family members, peers, health providers and health policymakers. Previous similar work by Wei in China,[36,37] and Dodd in the Philippines,[38] will inform this evaluation. The data will be analyzed using a thematic approach,[39] reported based on Consolidated Criteria for Reporting Qualitative Studies (COREQ).[40]

4.5 Cost-effective analysis: An incremental cost effectiveness analysis will be conducted to estimate the added benefits of the intervention regarding extra 1% of patients with blood pressure under control versus usual care from a societal perspective, which entails an assessment of all resource costs irrespective of payer. Costs associated with intervention over and above usual care (excluding research costs) will be collected and used together with the above effectiveness/outcome measures to calculate the incremental cost-effectiveness ratio.[40] Costs will include capital costs such as training, other costs to strengthen the capacity of providers to deliver enhanced services, and recurrent costs such as drugs and laboratory tests. Salaries of staff in RHCs will be included in case of extra working time is sought in some clusters during the intervention. Project record review and interviews with managers will be conducted to obtain cost data. Hospitalization and any loss of workdays due to hypertension, diabetes or COVID related illness, as well as time costs associated with participants' travel and waiting time will be recorded and converted into monetary forms using conventional age-sex adjusted market wages. We will use net benefit regression framework which will allow us to adjust for potential confounders and to calculate the clustered standard errors using the sandwich variance estimators to account for a clustered randomized design.[41] Markov models [42] will be used to simulate possible future states of the intervention, and to capture possible longer-term health outcomes (e.g. additional years of life) and associated costs of care.

4.6 Data collection, management, and analysis

We will collect patients' demographic and social information, their exact home address, blood pressures, body weight, and height, knowledge, attitude and practice (KAP) regarding NCD and COVID at the randomization and Month 10 in the RHCs. The RHC doctor will measure the participants' blood pressure using a calibrated electronic blood pressure apparatus after 5 minutes of seated rest, and measure body weight and height. Participants will be asked to provide a blood sample in the RHC at the recruitment and Month 10. In addition, we will also collect patient blood pressures in Month 10 using same methods. Blood samples will be collected and analyzed in RHC according to national guidelines for fasting blood glucose. We will collect attendance rates using the routine patient treatment cards in RHCs to record both physical and virtual consultations. We will conduct a survey at the recruitment to collect patient demographics, socioeconomics, smoking status, COVID-19 immunization status, health service use, medial, KAPs for NCD and COVID, and health care and related costs. The survey will be repeated at Month 10 in the last follow-up to collection information regarding COVID-19 symptoms and related health care consultation and use. Any deaths that occur in patient group during this period will be assigned a probable cause, as determined by a method called verbal autopsy.[43]

Overall, we will conduct mixed methods evaluation to examine the utility of the NCD-COVID guideline, the platforms and care process to inform acceptability, feasibility, reach, adoption, and implementation related challenges (in Section 5.2).

We will present appropriate descriptive statistics for RHCs and individual level, along with appropriate summary statistics and their associated 95% confidence intervals for all the outcomes. Then, for all outcomes, we will produce a main set of estimates of intervention effectiveness using cluster-level methods of analysis suitable for cluster trials. In particular, a linear mixed effect model will be used to analyse the primary outcome (systolic blood pressure) adjusting for the baseline systolic blood pressure. For other continuous secondary outcomes a similar analysis will be employed. Binary secondary outcomes will be analysed with the generalized estimating equation (GEE) approach, employing a binomial family with logit link function and exchangeable working correlation structure on sites. There will no interim analyses. Intent to treat analysis will be conducted.

4.4 Phase III: Evaluation and Knowledge Dissemination (Objective 3&4) (3 months): The phase III will also be used to complete the outcome evaluation, process and cost-effective evaluations highlighted in phase II. We will work together to 1) compile and analyze quantitative and qualitative data in Pakistan and Canada and produce reports and papers; and 2) coordinate with Department of Health Services at the provincial and national levels to translate evidence into program decisions for providing integrated COVID-NCD care in Pakistan and other LMICs. In addition, we will conduct four activities regarding the success factors for scaling up our interventions in LMICs:[44] 1) budget impact analysis: we will review the project costs, and estimate the annual costs for implementation which will inform the affordability in the health system; 2) develop a practical monitoring framework with multi-stakeholders, to inform its usability for future scale-up to other countries; 3) document factors that either facilitate or hinder scale-ups; and 4) provide detailed scale-up plans. The results of the implementation and scale-up will be communicated to policy makers by constantly engaging them in all the stages of the study using workshops, meetings and policy briefs. We will publish the results in leading academic journals and provide policy briefs to policymakers.

5. Project management: The investigators will seek ethical approvals from the Research Governance Committees in Canada and Pakistan (if needed). The study will be conducted to protect human rights and dignity of the participants. The study has been designed to minimize the burden of participants and any foreseeable risks in relation with the interventions. Interventions of our care program have no known harms. Starting from the usual care period, all participant patients will receive study information in Urdu and English and be invited to participate in the study by research staff in RHCs. We will collect written consents where feasible. We will collect informed consents from all participants. Dr. Wei and Dr. Khan will be co-guarantees who have full access to the trial dataset. A trial management unit (TMU) will be established in Islamabad, Pakistan with a trial manager and one staff locally and a research coordinator from UofT. TMU will manage the day-to-day activities of the trial. Dr. Thorpe will be the lead statistician, supported by Dr. Hicks. External member led DSMC will be established to safeguard the safety and privacy of patients involved, and to ensure that all data are collected according to agreed ethical guidelines, properly stored and only used for research purposes. A trial steering committee (TSC) will be established and led by external members. We will organise teleconference meetings for both the DSMC and TSC at the beginning of the study, and then on quarterly basis. The committees may also meet on an ad hoc basis should the need arise.

References

1. The Independent Panel. *Second report on progress by the Independent Panel for Pandemic Preparedness and Response for the WHO Executive Board, January 2021*. (2021).

2. Basu, S. & Phillips, R. S. *Primary Care in the COVID-19 Pandemic Improving access to high-quality primary care, accelerating transitions to alternative forms of care delivery, and addressing health disparities*. (2021).

3. Roth, G. A. *et al.* Global, regional, and national age-sex-specific mortality for 282 causes of death in 195 countries and territories, 1980–2017: a systematic analysis for the Global Burden of Disease Study 2017. *The Lancet* **392**, 1736–1788 (2018).

4. Sentongo, P., Ssentongo, A. E., Heilbrunn, E. S., Ba, D. M. & Chinchilli, V. M. Association of cardiovascular disease and 10 other pre-existing comorbidities with COVID-19 mortality: A systematic review and meta-analysis. *PLOS ONE* **15**, e0238215 (2020).

5. Thornton, J. Covid-19: how coronavirus will change the face of general practice forever. *BMJ* **368**, (2020).

6. WHO. COVID-19 significantly impacts health services for noncommunicable diseases. *The World Health Organization* https://www.who.int/news/item/01-06-2020-covid-19-significantly-impacts-health-services-for-noncommunicable-diseases (2020).

7. Monaco, A. *et al.* Digital Health Tools for Managing Noncommunicable Diseases During and After the COVID-19 Pandemic: Perspectives of Patients and Caregivers. *J Med Internet Res 2021;23(1):e25652 https://www.jmir.org/2021/1/e25652* **23**, e25652 (2021).

8. Monaghesh, E. & Hajizadeh, A. The role of telehealth during COVID-19 outbreak: A systematic review based on current evidence. *BMC Public Health* **20**, 1–9 (2020).

9. Ledwidge, M. *et al.* Natriuretic peptide-based screening and collaborative care for heart failure: the STOP-HF randomized trial. *JAMA* **310**, 66–74 (2013).

10. Greenhalgh, T., Koh, G. C. H. & Car, J. Covid-19: a remote assessment in primary care. *BMJ (Clinical research ed.)* **368**, (2020).

11. Anjana, R. M. *et al.* Acceptability and Utilization of Newer Technologies and Effects on Glycemic Control in Type 2 Diabetes: Lessons Learned from Lockdown. *Diabetes technology & therapeutics* **22**, 527–534 (2020).

12. K Shifat Ahmed, S. A. *et al.* Impact of the societal response to COVID-19 on access to healthcare for non-COVID-19 health issues in slum communities of Bangladesh, Kenya, Nigeria and Pakistan: results of pre-COVID and COVID-19 lockdown stakeholder engagements On behalf of the Improvin. *BMJ Global Health* **5**, 3042 (2020).

13. WHO. *Country Cooperation Strategy*. http://apps.who.int/gho/data/node.cco (2018).

14. Dawn. Clear signs 4th Covid wave is starting, warns Asad Umar - Pakistan - DAWN.COM. *Dawn.com* https://www.dawn.com/news/1634076 (2021).

15. Shafi, S. T. & Shafi, T. A survey of hypertension prevalence, awareness, treatment, and control in health screening camps of rural central Punjab, Pakistan. *Journal of epidemiology and global health* **7**, 135–140 (2017).

16. Basit, A., Fawwad, A., Siddiqui, S. A. & Baqa, K. Current management strategies to target the increasing incidence of diabetes within Pakistan. *Diabetes, Metabolic Syndrome and Obesity: Targets and Therapy* **12**, 85 (2019).

17. Mirco, N. *et al.* At the Epicenter of the Covid-19 Pandemic and Humanitarian Crises in Italy: Changing Perspectives on Preparation and Mitigation. *Catalyst: Innovations in Care Delivery* (2020) doi:10.1056/CAT.20.0080.

18. PTA. Telecom Indicators. *Pakistan Telecom Authority 2021* https://www.pta.gov.pk/en/telecom-indicators (2021).

19. Jordanova, M. & Lievens, F. *A Century of Telemedicine: Curatio Sine Distantia et Tempora A World Wide Overview-Part IV*. (2021).

20. Skegg, D. *et al.* Future scenarios for the COVID-19 pandemic. *Lancet (London, England)* **397**, 777–778 (2021).

21. Khan, M. A. *et al.* Enhanced hypertension care through private clinics in Pakistan: a cluster randomised trial. *BJGP Open* **3**, (2019).

22. Khan, M. A. *et al.* Effectiveness of an integrated diabetes care package at primary healthcare facilities: a cluster randomised trial in Pakistan. *BJGP Open* **2**, 1–11 (2018).

23. Arora, S. *et al.* Outcomes of treatment for hepatitis C virus infection by primary care providers. *The New England journal of medicine* **364**, 2199–2207 (2011).

24. Haldane, V. *et al.* Codevelopment of COVID-19 infection prevention and control guidelines in lower-middle-income countries: the "SPRINT" principles. *BMJ Global Health* **6**, 6406 (2021).

25. Otu, A. *et al.* Training health workers at scale in Nigeria to fight COVID-19 using the InStrat COVID-19 tutorial app: an e-health interventional study: *https://doi.org/10.1177/20499361211040704* **8**, 204993612110407 (2021).

26. Gaglio, B., Shoup, J. A. & Glasgow, R. E. The RE-AIM framework: a systematic review of use over time. *American journal of public health* **103**, (2013).

27. Glasgow, R. E., Vogt, T. M. & Boles, S. M. Evaluating the public health impact of health promotion interventions: the RE-AIM framework. *American journal of public health* **89**, 1322–1327 (1999).

28. Wei, X. *et al.* Implementation of a comprehensive intervention for patients at high risk of cardiovascular disease in rural China: A pragmatic cluster randomized controlled trial. *PloS one* **12**, (2017).

29. Wei, X. *et al.* Protocol for a randomised controlled trial to evaluate the effectiveness of improving tuberculosis patients' treatment adherence via electronic monitors and an app versus usual care in Tibet. *Trials* **20**, (2019).

30. Wei, X. *et al.* Effect of a training and educational intervention for physicians and caregivers on antibiotic prescribing for upper respiratory tract infections in children at primary care facilities in rural China: a cluster-randomised controlled trial. *The Lancet Global Health* **5**, e1258–e1267 (2017).

31. Khan, M. A. *et al.* Process evaluation of integrated diabetes management at primary healthcare facilities in Pakistan: a mixed-methods study. *BJGP open* **2**, 1–13 (2018).

32. Dure Technologies. About Dure Technologies. https://www.duretechnologies.com/#about (2022).

33. Walley, J. *et al.* Embedded health service development and research: Why and how to do it (a ten-stage guide). *Health Research Policy and Systems* **16**, 1–8 (2018).

34. Lee, Y. L., Lim, Y. M. F., Law, K. B. & Sivasampu, S. Intra-cluster correlation coefficients in primary care patients with type 2 diabetes and hypertension. *Trials* **21**, 1–10 (2020).

35. Lee, Y. L., Lim, Y. M. F., Law, K. B. & Sivasampu, S. Erratum: Intra-cluster correlation coefficients in primary care patients with type 2 diabetes and hypertension (Trials (2020) 21 (530) DOI: 10.1186/s13063-020-04349-4). *Trials* **21**, 1–1 (2020).

36. Wei, X. *et al.* Adapting a generic tuberculosis control operational guideline and scaling it up in China: A qualitative case study. *BMC Public Health* **8**, 1–10 (2008).

37. Wei, X. *et al.* Understanding factors influencing antibiotic prescribing behaviour in rural China: a qualitative process evaluation of a cluster randomized controlled trial. *Journal of health services research & policy* **25**, 94–103 (2020).

38. Lee, S. *et al.* The Presence of Cough and Tuberculosis: Active Case Finding Outcomes in the Philippines. *Tuberculosis research and treatment* **2019**, 1–9 (2019).

39. Patton, M. Q. Qualitative research and evaluation methods: Theory and practice. *SAGE Publications, Inc.* 832 (2015).

40. Tong, A., Sainsbury, P. & Craig, J. Consolidated criteria for reporting qualitative research (COREQ): a 32-item checklist for interviews and focus groups. *International journal for quality in health care : journal of the International Society for Quality in Health Care* **19**, 349–357 (2007).

41. Diggle, Peter. & Diggle, Peter. Analysis of longitudinal data. 379 (2002).

42. Sonnenberg, F. A. & Beck, J. R. Markov models in medical decision making: a practical guide. *Medical decision making : an international journal of the Society for Medical Decision Making* **13**, 322–338 (1993).

43. Jha, P. What is Verbal Autopsy? *Centre for Global Health Research* https://www.cghr.org/projects/million-death-study-project/what-is-verbal-autopsy/ (2021).

44. Milat, A. J., Bauman, A. & Redman, S. Narrative review of models and success factors for scaling up public health interventions. *Implementation Science* **10**, 1–11 (2015).
